# The value of noncoronary atherosclerosis for identifying coronary artery disease: results of the Leipzig LIFE Heart Study

**DOI:** 10.1007/s00392-015-0900-x

**Published:** 2015-09-11

**Authors:** Alexander Weissgerber, Markus Scholz, Andrej Teren, Marcus Sandri, Daniel Teupser, Stephan Gielen, Joachim Thiery, Gerhard Schuler, Frank Beutner

**Affiliations:** Department of Cardiology, Heart Center University Leipzig, Strümpellstr. 39, 04289 Leipzig, Germany; LIFE-Leipzig Research Center for Civilization Diseases, University Leipzig, Leipzig, Germany; Institute of Medical Informatics, Statistic and Epidemiology, University Leipzig, Leipzig, Germany; Institute of Laboratory Medicine, Ludwig-Maximilian University, Munich, Germany; Department of Medicine III, University Hospital Halle (Saale), Halle/Saale, Germany; Institute of Laboratory Medicine, Clinical Chemistry and Molecular Diagnostics, University Hospital Leipzig, Leipzig, Germany

**Keywords:** Coronary artery disease, Noncoronary atherosclerosis, carotid artery plaque, Intima-media thickness, Ankle-brachial index

## Abstract

**Background:**

Despite the widespread use of noninvasive testing prior to invasive coronary diagnostic the diagnostic yield of elective coronary angiography has been reported low in subjects with suspected obstructive CAD.

**Objective:**

To determine the predictive value of noncoronary atherosclerosis (NCA) in subjects with suspected stable coronary artery disease (CAD) intended to invasive coronary angiography.

**Methods:**

Ultrasound-based assessment of carotid artery plaque (CAP), carotid intima-media thickness (CIMT) and ankle-brachial index (ABI) was performed in 2216 subjects with suspected CAD prior to coronary angiography. Logistic regression and c-statistics were used to analyze the diagnostic value of NCA for the presence of obstructive CAD and the intention to revascularization.

**Results:**

Percentage of positive results of elective coronary angiography was low but comparable to other studies (41 % obstructive CAD). We identified 1323 subjects (60 %) with NCA, most of them were characterized by CAP (93 %). CAP independently predicted obstructive CAD in addition to traditional risk factors and clinical factors while CIMT and ABI failed to improve the prediction. The presence of NCA and typical angina were the strongest predictors for obstructive CAD (OR 4.0 and 2.4, respectively). A large subgroup of patients (*n* = 703, 32 %) with atypical clinical presentation and lack of NCA revealed a low indication for revascularization <15 % indicating a large proportion of subjects with non-obstructive CAD in this subgroup.

**Conclusion:**

The evaluation of noncoronary atherosclerosis has the potential to impact clinical decision making and to direct subsequent diagnostic procedures in subjects with suspected coronary artery disease.

**Clinical trial registration:**

NCT00497887.

**Electronic supplementary material:**

The online version of this article (doi:10.1007/s00392-015-0900-x) contains supplementary material, which is available to authorized users.

## Background

Noninvasive testing is recommended in patients with suspected stable ischemic heart disease (SIHD) prior to invasive coronary angiography to identify subjects at risk [1, 2]. Functional or stress testing to detect inducible ischemia has been the gold standard and is the most common noninvasive test used to diagnose SIHD. However, its high predictive value shown in myriad diagnostic studies is not translated into clinical reality. In less than half of the patients intended to elective coronary angiography significant obstructive CAD is confirmed [3]. The diagnostic yield of current noninvasive testing before heart catheterization is low and patients with a positive result on a noninvasive test exhibit only a slightly higher likelihood to have obstructive CAD than those who did not undergo any testing [3].

The generalized nature of atherosclerosis is expressed in the concomitant occurrence of CAD with noncoronary atherosclerotic diseases [4]. Noninvasive imaging tests aiming at detecting noncoronary atherosclerotic disease might be helpful in addition to the clinical presentation and functional/stress testing to direct diagnostic strategies in subjects with unknown but suspected CAD.

In the last two decades, measures of noncoronary atherosclerosis [NCA, e.g., carotid artery plaque, carotid intima-media thickness (CIMT), ankle-brachial index (ABI)] became surrogate marker broadly accepted for the prediction of incident cardiovascular events [5–7]. Recent studies showed that especially ultrasound-based carotid artery plaque assessment is also predictive for prevalent CAD [8–17]. The appropriate use of CIMT and ABI testing and its impact as measures for risk assessment have been defined for several scenarios [18, 19]. The clinical impact of the ultrasound-based assessment of noncoronary atherosclerosis in subjects with suspected CAD considered for invasive cardiac catheterization has not been defined so far.

In the present study, we investigate the association of carotid artery ultrasound measures (CIMT, carotid plaque), the ankle-brachial index (ABI) and the patients history of peripheral or cerebrovascular disease with obstructive CAD as confirmed by coronary angiography in subjects with suspected CAD intended for invasive coronary diagnostic.

## Methods

### Study cohort

The Leipzig Heart Study is designed as an observational study to evaluate biomarkers and their ability to assess the presence and severity of CAD in subjects with suspected CAD. A detailed description of study design and baseline characteristics has been published elsewhere [20]. The study meets the ethical standards of the Declaration of Helsinki, and written informed consent was obtained from all participants.

The present study included 2552 subjects with suspected stable CAD. Patients were admitted to the Leipzig University Heart Center (2006–2011) for invasive coronary angiography due to the presence of clinical symptoms (e.g., chest pain, shortness of breath) and positive noninvasive testing (e.g., cardiopulmonary exercise test, echocardiographic, nuclear or magnetic resonance imaging). Patients with known CAD and any previous coronary revascularization in form of percutaneous coronary intervention (PCI) or coronary artery bypass graft (CABG) were excluded in order to recruit only those with first onset of symptoms and untreated coronary arteries. We excluded 195 cases in which documentation of their medical history was incomplete and 141 cases in which ultrasound was not available or with insufficient quality. Finally, 2216 subjects were included into the analyses.

### Risk factors and clinical presentation

Risk factors and clinical presentation were obtained from a standardized interview, a standardized biometric assessment and laboratory analyses as published before [20]. Chest pain was inquired using the WHO Rose Angina Questionnaire [21]. The classification of chest pain in nonanginal chest pain, atypical angina and typical angina was performed according to the guidelines [1]. High-sensitive C-reactive protein (hs-CRP) and n-terminal pro brain natriuretic peptide (nt-proBNP) have been shown associated with cardiovascular phenotypes and coronary events in stable subjects and were integrated in the analyses [22–25].

### Evaluation of noncoronary atherosclerosis

#### History of peripheral vascular disease (PVD)

Information about interventions due to lower extremity artery disease (LEAD) and cerebrovascular disease (CVD) were obtained from the interview.

#### Carotid artery plaque

The ultrasound procedure of carotid arteries has been described in detail before [20] (brief description in supplemental material). Carotid artery plaque was defined as recommended by the American Society of Echocardiography Intima-Media Thickness Task Force [26]: echogenic thickening of intimal reflection that extends into the arterial lumen at least 0.5 mm or 50 % of the surrounding CCA-IMT value or an intimal + medial thickness of >1.5 mm. Plaque presence was documented as ‘present’ or ‘absent’ for the common part and bulb of the right and left carotid artery, respectively. A simple plaque score (PS) was calculated by counting segmental plaque presence of the common carotid artery and bulb. As the extracranial length of internal carotid artery and the quality of its imaging is variable, we restricted plaque score determination to the common part and the bulb resulting in values of 0–4. Intra- and inter-reader reliability of carotid artery plaque assessment were tested in scans of 60 subsequent subjects being read by 4 sonographers, each blinded from the other’s findings. Krippendorff’s alpha was 0.90 for intra-reader reliability and 0.65 for interreader reliability (further data in Supplemental Table 1).

#### Carotid intima-media thickness (CIMT)

The mean and maximum of the combined thickness of the intimal and medial layer of the far wall of the CCA were measured with a semiautomated border detection program (EchoPAC Dimension 06, GE Medical Systems, Munich, Germany). The detecting area of CIMT was defined as the distal 1 cm (about 250 single measure points) of the common carotid arteries, proximal to the origin of the bulb (Supplemental Fig. 2a–c). Measurements of the left and right sides were averaged to obtain the CIMTmean, the higher value of the left and right maximum CIMT was used to obtain CIMTmax. Intra- and inter-reader reliability of CIMT were tested in scans of 60 subsequent subjects being read by 4 sonographers, each blinded from the other’s findings. Concordance correlation coefficients (CCC) for intra-reader reliability were 0.95 (CIMTmean) and 0.91 (CIMTmax), CCC for interreader reliability were 0.90 (CIMTmean) and 0.87 (CIMTmax), further data in the Supplemental Table 1.

#### Ankle-brachial index (ABI)

Systolic blood pressures of the right arm and both ankles are measured twice by gold standard method Doppler ultrasound using sphygmomanometer cuffs and a handheld Doppler probe (Huntleigh Mini-Dopplex, Kempen, Germany) [27]. ABI was calculated by dividing the averaged systolic blood pressure at the posterior tibial artery by the averaged systolic blood pressure in the arm. The lower ABI of the right and left side was used for analyses. Subjects were categorized in subgroups of normal (≥1.1), low-normal (1.00–1.09), borderline (0.9–0.99), pathological (0.5–0.89) and critical (<0.5) ABI.

### Coronary angiography

Coronary angiography was performed following the standards of our institution [20]. Patients were classified in subsets with (1) normal angiogram or nonobstructive CAD with luminal reduction <50 % and (2) obstructive CAD with at least one stenosis ≥50 % in a major coronary vessel. Additionally, subjects were classified with respect to subsequently induced therapeutic interventions namely conservative therapy or intention to coronary revascularization [percutaneous coronary intervention (PCI) or coronary artery bypass grafting (CABG)].

### Statistics

Categorical data are presented as numbers or percentages and were compared using *χ*^2^ or Fisher’s exact test, as appropriate. For continuous variables, we present arithmetic mean ± standard deviation for normally distributed variables and median ± standard deviation for non-normally distributed variables. Continuous variables were compared using Student’s *t* test and Mann–Whitney *U* test, as appropriate. Odds ratios (OR) of CIMTmean, CIMTmax (per 0.1 mm), carotid artery plaque (per point of plaque score), ABI and clinical factors were obtained from logistic regression analyses, adjusted for traditional risk factors (age, gender, diabetes, tobacco use, hypertension, dyslipidemia). Stepwise forward logistic regression (Wald Chi square) was performed to identify major predictors. Receiver operating characteristic (ROC) was performed for:

1. Traditional risk factors (TRF),
2. TRF + clinical factors [clinical presentation (angina, NYHA), history of PVD, reduced left-ventricular ejection fraction <50 %, hsCRP, ntproBNP] and the incremental impact of carotid ultrasound measures to 2):
3. +CIMT_mean_,
4. +Carotid artery plaque,
5. +ABI.

Sensitivity and specificity were calculated for a positive carotid plaque test (≥1 plaque in CCA or bulb). *P* values <0.05 were considered statistically significant. IBM SPSS Statistics 20 was used for statistical analyses of models. The statistical software package “R” (www.r-project.org”) was used to perform concordance analyses.

## Results

### Baseline characteristics of the participants

Demographic and clinical characteristics of the study subjects are summarized in Table 1. The median age was 64 years (interquartile range 54–70 years); 35.3 % were female; 31.7 % were diabetics. Chest pain was the reason for cardiac diagnostic assessment in 64.2 % of participants. 25.0 % presented with typical angina and 39.2 % were classified as atypical angina or non-cardiac chest pain. The remaining subjects presented with other atypical clinical presentation but a positive result in former noninvasive testing. The distribution of the clinical presentation in our German cohort intended for invasive coronary diagnostic is comparable with the American NCDR CathPCI Registry. In this large-scale study, 33 % subjects were graded as stable angina and the remaining subjects with atypical or asymptomatic presentation [3].

**Table 1:** Baseline characteristics of the study participants

Characteristic	Total (*n* = 2216)	Obstructive CAD (*n* = 913)	No obstructive CAD (*n* = 1303)	*P* value
Age (years)	64 ± 11	66 ± 11	62 ± 11	<0.001
Female sex (%)	35.3	23.5	43.5	<0.001
Body mass index (kg/m^2^)	29.2 ± 5.0	29.0 ± 4.6	29.4 ± 5.2	0.190
Waist-hip ratio	0.98 ± 0.08	1.01 ± 0.07	0.96 ± 0.09	<0.001
Diabetes (%)	31.7	35.9	28.7	<0.001
Hypertension (%)	83.3	84.1	82.7	0.387
Dyslipidemia (%)	47.8	58.3	41.7	<0.001
Tobacco use				
Former (%)	38.3	42.4	35.5	<0.001
Current (%)	17.0	20.4	14.7	
Family history (%)	32.4	33.1	31.8	0.553
Ejection fraction <50 %	15.3	19.5	12.3	<0.001
hsCRP (mg/l)	2.2 ± 9.7	2.6 ± 11.8	2.0 ± 7.7	<0.001
Nt-proBNP (ng/l)	149 ± 1255	226 ± 1480	120 ± 1056	<0.001
Medication				
ASA (%)	50.6	59.5	44.0	<0.001
Beta blocker (%)	57.9	58.4	57.6	0.783
RAAS antagonist (%)	67.6	69.3	66.3	0.293
Calcium antagonist (%)	24.0	24.5	23.6	0.717
Diuretic (%)	38.1	38.4	37.8	0.849
Statin (%)	36.6	42.7	32.2	<0.001
Clinical presentation				
NCP/AA/TA (%)	15.7/23.5/25.0	11.0/22.0/35.0	19.1/24.6/18.0	<0.001
NYHA II/III (%)	41.4/11.0	40.4/9.4	42.1/12.3	0.009

*P* value refers to the comparison of the obstructive CAD/no obstructive CAD groups

*hsCRP* high-sensitive C-reactive protein, *NCP* nonanginal chest pain, *AA* atypical angina, *TA* typical angina. *P* value refers to the comparison of the obstructive CAD/no obstructive CAD groups

### Prevalence of noncoronary atherosclerosis

Findings of noncoronary atherosclerosis are summarized in Table 2. History of peripheral artery disease was reported in 4.5 % of the subjects. Carotid arterial lesions were present in 55.7 % (men 61.8 %, women 44.1 %). Carotid artery plaque more often occurs in the bulb (54.0 %) than in the common part of the carotid artery (24.0 %, *P* < 0.001). The majority of subjects had a normal and low-normal ABI (51.4 and 29.7 %, respectively). Significant ABI values <0.9 were found in 254 subjects (11.5 %).

**Table 2:** Prevalence of noncoronary atherosclerosis

Characteristic	Total	Obstructive CAD	No obstructive CAD	*P* value
History of atherosclerotic disease				
LEAD (%)	3.4	5.9	1.7	<0.001
CVD (%)	1.3	2.0	0.8	0.022
PVD (%)	4.5	7.3	2.5	<0.001
Presence of carotid arterial lesions				
Carotid plaque (%)	55.7	73.9	43.0	<0.001
CPS 1/2/3/4 (%)	16.8/19.9/10.2/8.9	17.9/25.1/15.7/15.2	16.0/16.2/6.3/4.5	<0.001
CIMT_mean_ (mm)	0.78 ± 0.15	0.82 ± 0.15	0.76 ± 0.14	<0.001
CIMT_max_ (mm)	0.94 ± 0.20	0.99 ± 0.21	0.92 ± 0.19	<0.001
Presence of lower extremity arterial disease				
ABI ≥ 1.1	51.4	43.8	56.7	<0.001
1.0–1.09	29.7	27.5	31.3	
0.9–0.99	7.4	9.9	5.7	
0.5–0.89	9.3	14.5	5.6	
<0.5	2.2	4.4	0.7	
Cumulative NCA (%)	59.7	78.1	46.8	<0.001

*P* value refers to the comparison of the obstructive CAD/no obstructive CAD groups

*LEAD* lower extremity artery disease, *CVD* cerebrovascular disease, *PVD* peripheral vascular disease, *CPS* carotid plaque score, *CIMT* carotid intima-media thickness, *ABI* ankle-brachial index, *NCA* noncoronary atherosclerosis

Cumulative noncoronary atherosclerotic disease was identified in 1323 subjects (59.7 %). Thereof, carotid artery plaque was present in the majority of subjects (93.3 %), the other PVD characteristics were less present (PVD in history 7.5 %, CIMT_max_ > 1.5 mm 2.3 %, ABI < 0.9 19.2 % of subjects with NCA).

### Prevalence of coronary artery disease

Angiographically significant CAD was present in 41.2 % of the subjects (48.7 % of men, 27.5 % of women) resulting in assignment to PCI or CABG in 37.5 % of males and 20.8 % of females. The remaining subjects were free of angiographically visible CAD or showed wall irregularities <50 % luminal reduction. These findings of diagnostic angiography show high accordance with the American NCDR CathPCI Registry (41.0 % with obstructive CAD) [3].

### Predictors of obstructive CAD

Known conventional risk factors higher age, male sex, diabetes, dyslipidemia and tobacco use were independently associated with obstructive coronary artery disease and revascularization. An echocardiographic reduction of the left-ventricular ejection fraction, higher levels of high-sensitive C-reactive protein and nt-pro brain natriuretic peptide were also independently associated with obstructive coronary artery disease (Table 3) and revascularization (Supplemental Table 2).

**Table 3:** The value of traditional risk factors and clinical characteristics for predicting obstructive CAD

Variable	Unadj. odds ratio (95 % CI)	*P* value	Adj. odds ratio (95 % CI)	*P* Value
Traditional risk factors				
Age, per 5 years increase	1.13 (1.08–1.17)	<0.001		
Male sex	2.50 (2.07–3.02)	<0.001		
Diabetes	1.39 (1.16–1.66)	<0.001		
Dyslipidemia	1.95 (1.64–2.31)	<0.001		
Hypertension	1.11 (0.88–1.39)	0.389		
Former tobacco use	1.60 (1.32–1.93)	<0.001		
Current tobacco use	1.86 (1.46–2.37)	<0.001		
Additional clinical characteristics				
Family history of CAD	1.06 (0.87–1.30)	0.553	1.19 (0.96–1.48)	0.118
LV-EF <50 %	1.73 (1.37–2.19)	<0.001	1.31 (1.02–1.69)	0.034
hsCRP, per 5 mg/l	1.11 (1.06–1.17)	<0.001	1.08 (1.03–1.14)	0.004
Nt-proBNP, per 500 ng/l	1.11 (1.06–1.16)	<0.001	1.06 (1.01–1.10)	0.009
Clinical presentation				
Nonanginal chest pain	0.69 (0.52–0.90)	0.007	0.85 (0.64–1.14)	0.290
Atypical angina	1.07 (0.86–1.35)	0.543	1.38 (1.08–1.76)	0.011
Typical angina	2.33 (1.86–2.91)	<0.001	3.09 (2.42–3.95)	<0.001
Dyspnea NYHA II	0.83 (0.68–0.99)	0.042	0.90 (0.73–1.09)	0.274
NYHA III	0.67 (0.50–0.90)	0.008	0.64 (0.46–0.88)	<0.001

The clinical presentation of typical angina (OR 3.1) as well as atypical angina (OR 1.4) was associated with obstructive CAD and revascularization. In contrast, the classification ‘nonanginal chest pain’ and shortness of breath neither did predict obstructive CAD nor revascularization.

**Fig. 1:**
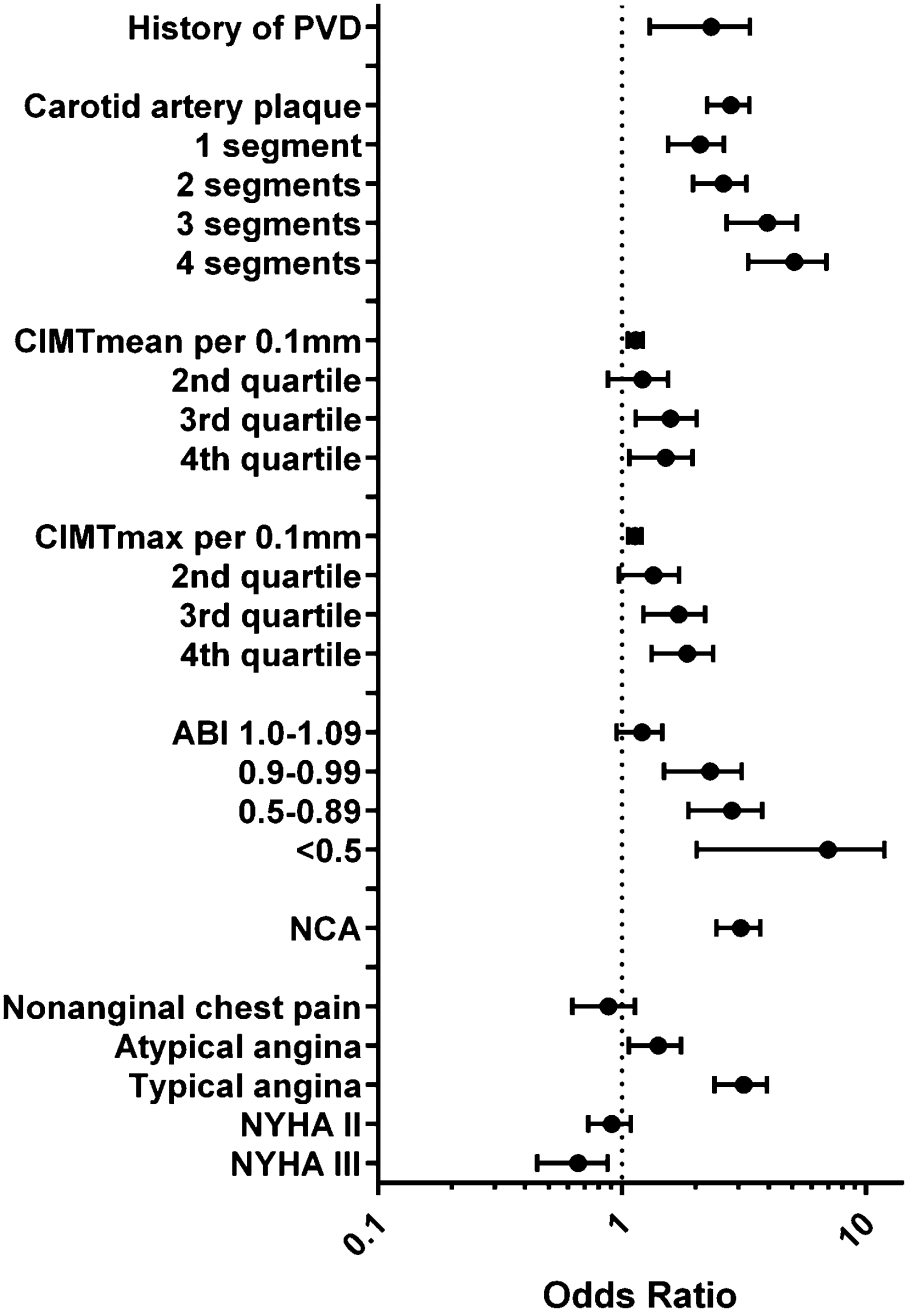
Odds ratios and error bars for measures of noncoronary atherosclerosis and features of clinical presentation predicting obstructive CAD in subjects with suspected CAD

All noncoronary atherosclerotic disease characteristics were independently (i.e., after adjustment for traditional risk factors) associated with obstructive coronary artery disease (Fig. 1; Table 4) and revascularization (Supplemental Table 2). The presence of carotid artery plaque predicted obstructive CAD with OR 2.8. Multisegmental affection of the carotid arterial system linearly increased the predictive value to OR 4.9. Mean and maximum CIMT were independently associated with CAD (per 0.1 mm increase OR 1.13). ABI values <1.0 predicted obstructive CAD with OR 2.5. In summary, any noncoronary atherosclerosis predicted obstructive CAD and revascularization with OR 3.0.

**Table 4:** The value of noncoronary atherosclerosis for predicting obstructive CAD

Variable	Unadj. odds ratio (95 % CI)	*P* value	Adj. odds ratio (95 % CI)	*P* value
History of PVD	3.14 (2.05–4.83)	<0.001	2.17 (1.38–3.41)	0.001
Carotid artery plaque	3.76 (3.13–4.52)	<0.001	2.75 (2.26–3.35)	<0.001
1 Segment	2.43 (1.89–3.13)	<0.001	2.04 (1.57–2.64)	<0.001
2 Segments	3.39 (2.67–4.30)	<0.001	2.54 (1.98–3.27)	<0.001
3 Segments	5.48 (4.03–7.46)	<0.001	3.81 (2.75–5.27)	<0.001
4 Segments	7.48 (5.33–10.5)	<0.001	4.87 (3.40–6.98)	<0.001
CIMT_mean_ per 0.1 mm	1.31 (1.23–1.40)	<0.001	1.13 (1.06–1.22)	0.001
CIMT_max_ per 0.1 mm	1.28 (1.21–1.35)	<0.001	1.13 (1.06–1.20)	<0.001
ABI 1.0–1.09	1.14 (0.93–1.39)	0.207	1.19 (0.96–1.47)	0.111
0.9–0.99	2.25 (1.61–3.13)	<0.001	2.20 (1.54–3.14)	<0.001
0.5–0.89	3.34 (2.45–4.56)	<0.001	2.71 (1.93–3.80)	<0.001
<0.5	8.21 (3.94–17.1)	<0.001	5.80 (2.71–12.4)	<0.001
ABI <1.0	2.96 (2.37–3.69)	<0.001	2.48 (1.95–3.16)	<0.001
NCA	4.05 (3.35–4.90)	<0.001	3.02 (2.46–3.71)	<0.001

### Diagnostic benefit of NCA testing

**Fig. 2:**
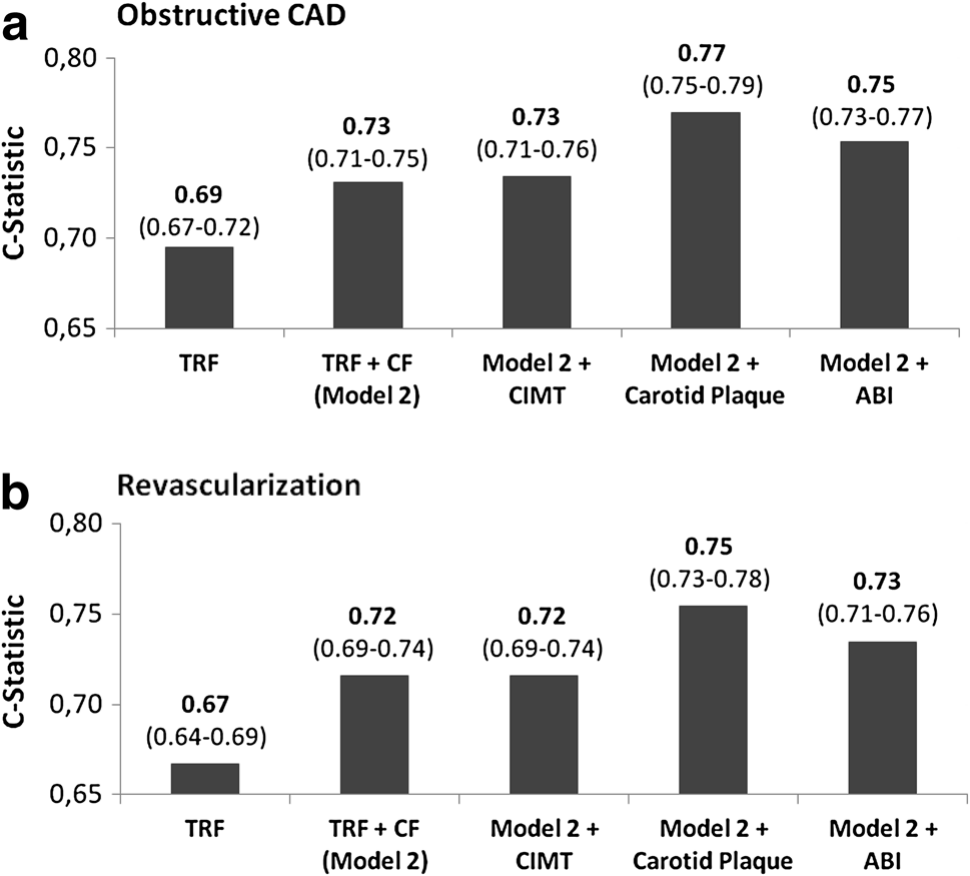
*Bars* indicate the area under the ROC curve of various models to predict obstructive CAD (**a**) and coronary revascularization (**b**). The simplest model (TRF) includes the traditional risk factors (age, gender, diabetes status, hypertension, tobacco use, serum LDL-cholesterol, HDL-cholesterol adjusted for lipid lowering medication). In model 2 further clinical factors (angina, NYHA, history of PVD, left-ventricular ejection fraction) and biomarker (hsCRP, ntproBNP) were included. Finally, vascular ultrasound measures (CIMT, carotid artery plaque, ABI) were added to model 2, respectively

To investigate which method of NCA testing is best suited to improve diagnostic of CAD and revascularization, we analyzed C-statistics of carotid plaque, ABI and CIMT. C-statistic showed that carotid artery plaque assessment best improved the prediction of obstructive CAD and the intention for revascularization while ABI was inferior and CIMT failed to give independent impact to a model including TRF and clinical factors (CF, including angina, NYHA, history of PVD, reduced left-ventricular ejection fraction <50 %, hsCRP, ntproBNP), Fig. 2. The area under the curve (AUC) to predict obstructive CAD improved from 0.69 (TRF) and 0.73 (TRF + CF) to 0.77 (TRF + CF + carotid plaque), *P* < 0.001. The AUC to predict the need for revascularization improved from 0.67 (TRF) and 0.72 (TRF + CF) to 0.75 (TRF + CF + carotid plaque), *P* < 0.001.

Calculating the optimal cut-off for the number of carotid artery segments diseased to predict obstructive CAD, Youden’s index reached its maximum at one segment diseased. The strength of NCA testing is the high negative predictive value especially in subjects with atypical clinical presentation reaching negative predictive values of 81 % for obstructive CAD and 87 % for revascularization (detailed data of specificity, sensitivity, positive and negative predictive value in Supplemental Table 3).

### Typical angina and noncoronary atherosclerosis—the major signs of stable obstructive CAD

**Fig. 3:**
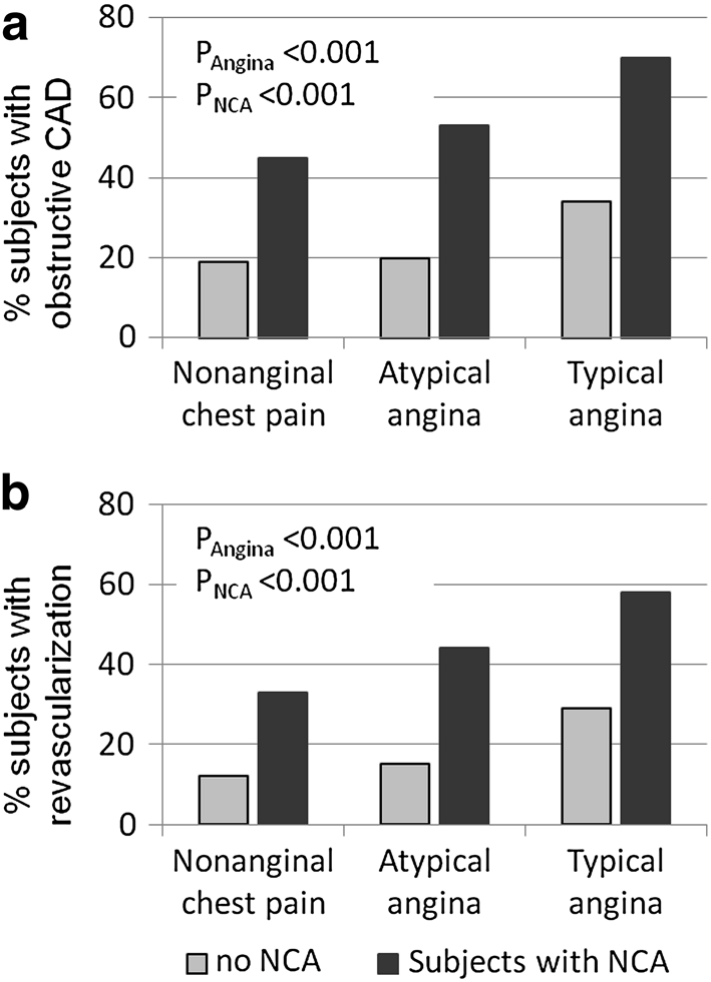
Percentage of subjects with obstructive CAD (**a**) and coronary revascularization (**b**) depending on the absence (*lightly colored*) and presence (*dark colored*) of noncoronary atherosclerotic disease (NCA) and the clinical presentation (x-axis, subset of nonanginal chest pain includes subjects without chest pain)

Stepwise logistic regression identified two major predictors for stable obstructive CAD: the presence of noncoronary atherosclerosis (OR 4.0, CI 3.3–4.8) and typical angina (OR 2.4, CI 1.9–3.0). Both were also the strongest predictors for revascularization: NCA OR 3.6 (CI 2.9–4.5) and typical angina OR 2.7 (CI 2.1–3.5). The impact of the assessment of NCA to predict CAD and revascularization was evident independently of age and gender (Supplemental Table 4). The strong predictive value of NCA was demonstrated independently of the clinical presentation (Fig. 3). However, the relevance of noncoronary atherosclerosis is pivotal in subjects with atypical clinical presentation. While subjects with typical angina already have a higher pretest probability for obstructive CAD and revascularization, subjects with atypical clinical presentation demonstrated a lower pretest probability with questionable necessity for invasive coronary angiography. Under this condition, the detection of noncoronary atherosclerosis significantly increased the pretest probability to higher than 40 %, while subjects without detectable noncoronary atherosclerosis were unlikely to have obstructive CAD (<20 %) and were even more unlikely to get coronary revascularization (<15 %, Fig. 3). Atypical clinical presentation and lacking detection of NCA applies to a large group of patients in our cohort (*n* = 703, 32 %).

## Discussion

The objective of our study was to evaluate the predictive value of noncoronary atherosclerosis assessment for identifying coronary artery disease and the need for revascularization in a cohort of patients with clinical symptoms indicating first-time elective invasive coronary angiography. Our major results are:

1. A simple ultrasound-based screening for noncoronary atherosclerosis improved the pretest probability of obstructive CAD and the intention to revascularization prior to invasive coronary angiography. Its strength is the high negative predictive value in subjects with atypical clinical presentation identifying a large group of subjects with low probability of obstructive CAD.
2. Especially ultrasound-based carotid artery plaque assessment was identified as a useful measure of noncoronary atherosclerosis. The presence of carotid artery plaque and its extent were independently associated with the first-time diagnosis of stable obstructive CAD after adjustment for traditional cardiovascular risk factors and further CAD-associated factors such as clinical presentation, known noncoronary vascular disease, reduced left-ventricular ejection fraction and additional biochemical markers.
3. CIMT and ABI also independently predicted obstructive CAD after adjustment for traditional risk factors but CIMT and ABI failed to add significant diagnostic value to a model including of established risk factors and carotid artery plaque.

The observation that carotid artery plaque is able to predict prevalent CAD has been reported in some small angiographic studies [11–17]. Our much larger study additionally investigated the relationship between carotid ultrasound findings and the intention for coronary revascularization. Furthermore, our analyses were interpreted in the context of important clinical factors including the clinical presentation, cardiac function and biochemical biomarkers which were disregarded in most of the previous studies.

Although typical angina is the major clinical presentation of stable CAD, and, as also demonstrated in our study, one of the strongest predictors of obstructive CAD, only a quarter to a third of subjects intended for elective coronary angiography due to suspected obstructive CAD presents with typical angina. The majority of patients have an atypical clinical presentation resulting in a high chance of negative angiographic results. For these patients, the evidence of noncoronary atherosclerosis is the most important predictor of obstructive CAD. Conversely, in the subset of subjects with atypical clinical presentation and no detectable noncoronary atherosclerosis the probability of obstructive CAD is low (<20 %) and the rate of coronary revascularization is even lower (<15 %). Interestingly, this applies to a relatively large group of patients in our cohort, about one third of patients. When cardiologists and other clinicians consider whether a patient with atypical presentation has significant coronary artery disease, they are less likely to want to determine whether noncoronary atherosclerosis disease is present, unless there is a strong suspicion that the presence of severe disease could determine the patient’s near future prognosis, before deciding on carrying out coronary angiography. However, the clinicians should consider the increased likelihood of CAD in the type of patients included in this study if there is evidence of noncoronary atherosclerosis and the non-invasive tests, particularly for the presence of carotid artery disease, are positive.

The superiority of carotid artery plaque assessment compared to CIMT is explained by its carotid-wide screening area, while CIMT measurement is limited to the far wall of the common part of the carotid arteries. Indeed, atherosclerotic disease occurs predominantly downstream to the region of CIMT measurement namely in the bulb and the proximal parts of the internal/external branches of the carotid artery [28]. We did not consider IMT measurements of other carotid artery regions due to the limited standardization of the measurements and focused on that recommended by the Carotid Intima-Media Thickness Task Force of the American Society of Echocardiography [26]. In line with our results, a recent meta-analysis showed that carotid artery plaque has a higher diagnostic accuracy compared with CIMT for the prediction of future CAD events [9]. Further studies also showed that CIMT added only little predictive value to conventional risk factors [8, 9, 11]. Today there is strong evidence for the superiority of carotid artery plaque assessment for the prediction of incident cardiovascular events, and as here shown also for prevalent CAD, which evokes increasing debate regarding the IMT paradigm [29–31].

A limitation of our study is the mono-centric design which might be biased by local characteristics of allocation and accomplishment. However, both the distribution of clinical presentation and angiographic findings is comparable with the representative large-scale American NCDR CathPCI Registry including hundreds of angiographic centers [3]. Second, we did not quantify carotid artery plaque size in this initial study because our results show that the optimal Youden’s index calculated from our semiquantitative plaque score was already reached when only one carotid artery segment was affected. This indicates that the presence of atherosclerosis per se is of importance. However, the fact that multi-locus carotid artery plaque further increase the risk of CAD points towards the usefulness of a more detailed quantification of carotid atherosclerosis. Several ongoing studies announced the application of three-dimensional carotid ultrasound to improve the prediction of the incident cardiovascular risk [10, 32]. Our carotid ultrasound approach would also allow 3D-morphometric quantification; we aim at improving CAD risk prediction on the basis o these measures in the future.

## Conclusion

Our study accentuates the diagnostic value of an assessment of noncoronary atherosclerosis, in particular that of carotid artery plaque, to improve the diagnostic yield of invasive coronary angiography in subjects with suspected coronary artery disease. The strength of carotid artery plaque assessment is pivotal in subjects with atypical clinical presentation, which represents the majority of subjects intended to diagnostic coronary angiography. In view of the low pretest probability within the large group of subjects with atypical clinical presentation and lack of noncoronary atherosclerosis, indication for invasive coronary diagnostic should be reassessed carefully. We conclude that carotid artery plaque detection has the potential to assess prognosis and to direct clinical decision making and subsequent diagnostic procedures in subjects with suspected coronary artery disease.

### Source of funding

Initial funding of the Leipzig Heart Study was supported by the Roland-Ernst Foundation; continuation is supported by LIFE-Leipzig Research Center for Civilization Diseases, University Leipzig. LIFE is funded by the Free State of Saxony within the framework of its excellence initiative.

## Electronic supplementary material

Supplementary material 1 (DOCX 2670 kb)
