# Supplementary material for: The value of noncoronary atherosclerosis for identifying coronary artery disease: results of the Leipzig LIFE Heart Study
Source: Clin Res Cardiol. 2015 Sep 11;105:172–81. doi: 10.1007/s00392-015-0900-x (PMC4735267; doi:10.1007/s00392-015-0900-x)
Supplement: Supplementary file 1 — Supplementary material 1 (DOCX 2670 kb) [file 392_2015_900_MOESM1_ESM.docx]

**Supplemental Material**

**The Value of Noncoronary Atherosclerosis for Coronary Artery Disease: Results of the Leipzig LIFE Heart Study**

Weissgerber A et al.

***Carotid artery ultrasound***

Ultrasonographic examination of the carotid arteries followed the recommendations of the American Society of Echocardiography[^1^](#_ENREF_1). Examinations are performed and interpreted by experienced and trained sonographers. High resolution B-mode ultrasound images are acquired using the GE Vivid 7 and Vivid q ultrasound platform with a 12.0-MHz linear-array transducer (GE Healthcare, Munich, Germany). Images are stored in raw data format for retrospective reading and measurement. Longitudinal views and transversal carotid artery sweeps are used to acquire images of the common, bulb and internal segments of each carotid artery (Supplemental Figure 1).


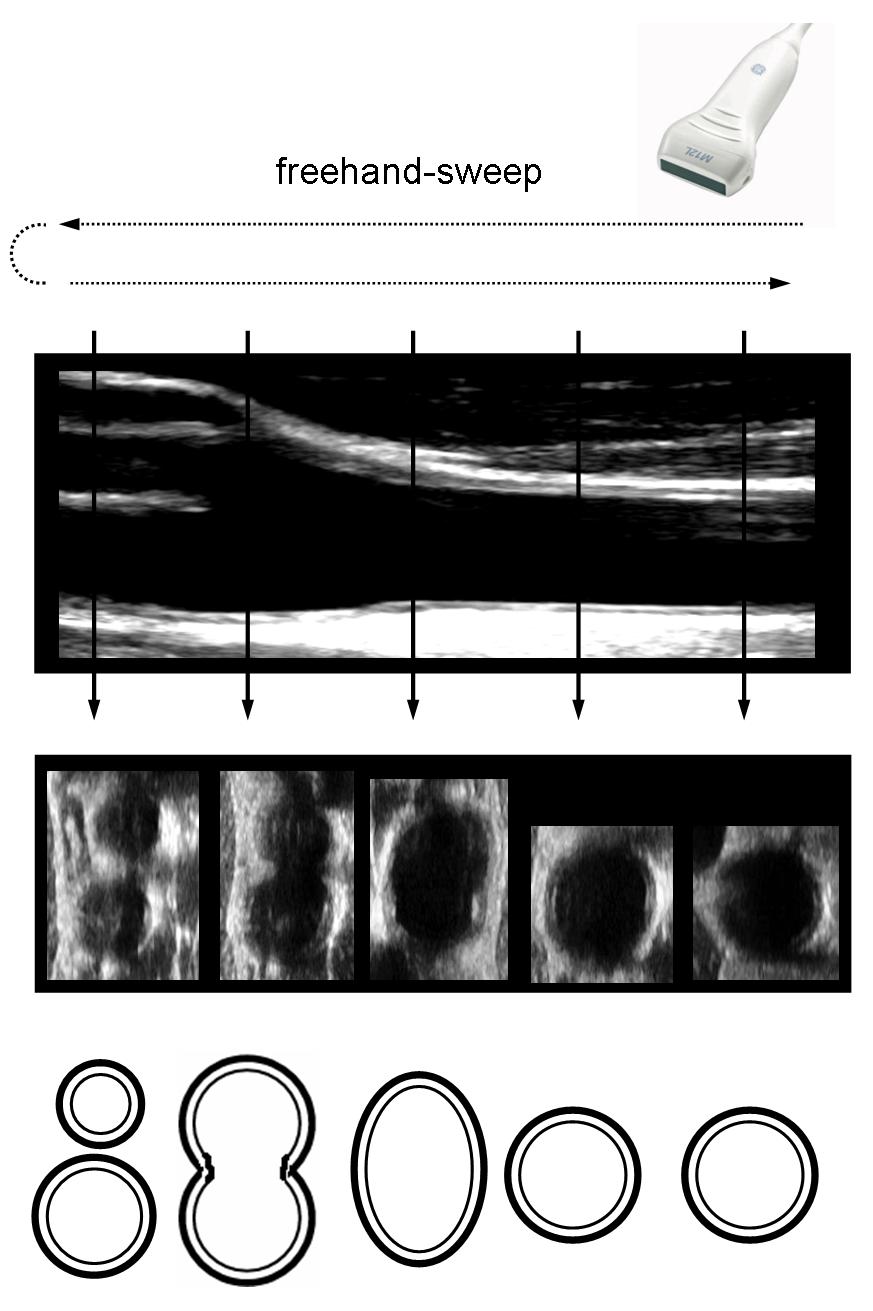


Supplemental Figure 1

Analysis followed the recommendations of the American Society of Echocardiography with separate categorization of CIMT and carotid artery plaque[^1^](#_ENREF_1).

***CIMT:*** The mean and maximum of the combined thickness of the intimal and medial layer of the far wall of the CCA were measured with a semiautomated border detection program (EchoPAC Dimension 06, GE Medical Systems, Munich, Germany). The detecting area of CIMT was defined as the distal 1 cm (about 250 single measure points) of the common carotid arteries, proximal to the origin of the bulb (Supplemental Figure 2, A-C). Measurements of the left and right sides were averaged to obtain the CIMTmean, the higher value of the left and right maximum CIMT was used to obtain CIMTmax.

Intra- and interreader reliability of CIMT were tested in scans of 60 subsequent subjects being read by 4 sonographers. Concordance correlation coefficients (CCC) for intrareader reliability were 0.95 (CIMTmean) and 0.91 (CIMTmax), CCC for interreader reliability were 0.90 (CIMTmean) and 0.87 (CIMTmax), further data in the Supplemental Table 1.

***Carotid artery plaque:*** CAP was defined as recommended by the American Society of Echocardiography Intima-Media Thickness Task Force: echogenic thickening of intimal reflection that extends into the arterial lumen at least 0.5 mm or 50% of the surrounding CCA-IMT value or an intimal + medial thickness of >1.5 mm. Plaque presence was documented as ‘present’ or ‘absent’ for the common part and bulb of the right and left carotid artery, respectively. A simple plaque score (PS) was calculated by counting segmental plaque presence of the common carotid artery and bulb. As the extracranial length of internal carotid artery and the quality of its imaging is variable, we restricted plaque score determination to the common part and the bulb resulting in values of 0 to 4.

Intra- and interreader reliability of CAP assessment were tested in scans of 60 subsequent subjects being read by 4 sonographers, each blinded from the other’s findings. Krippendorff’s alpha was 0.90 for intra-reader reliability and 0.65 for inter-reader reliability (further data in Supplemental Table 1).

**
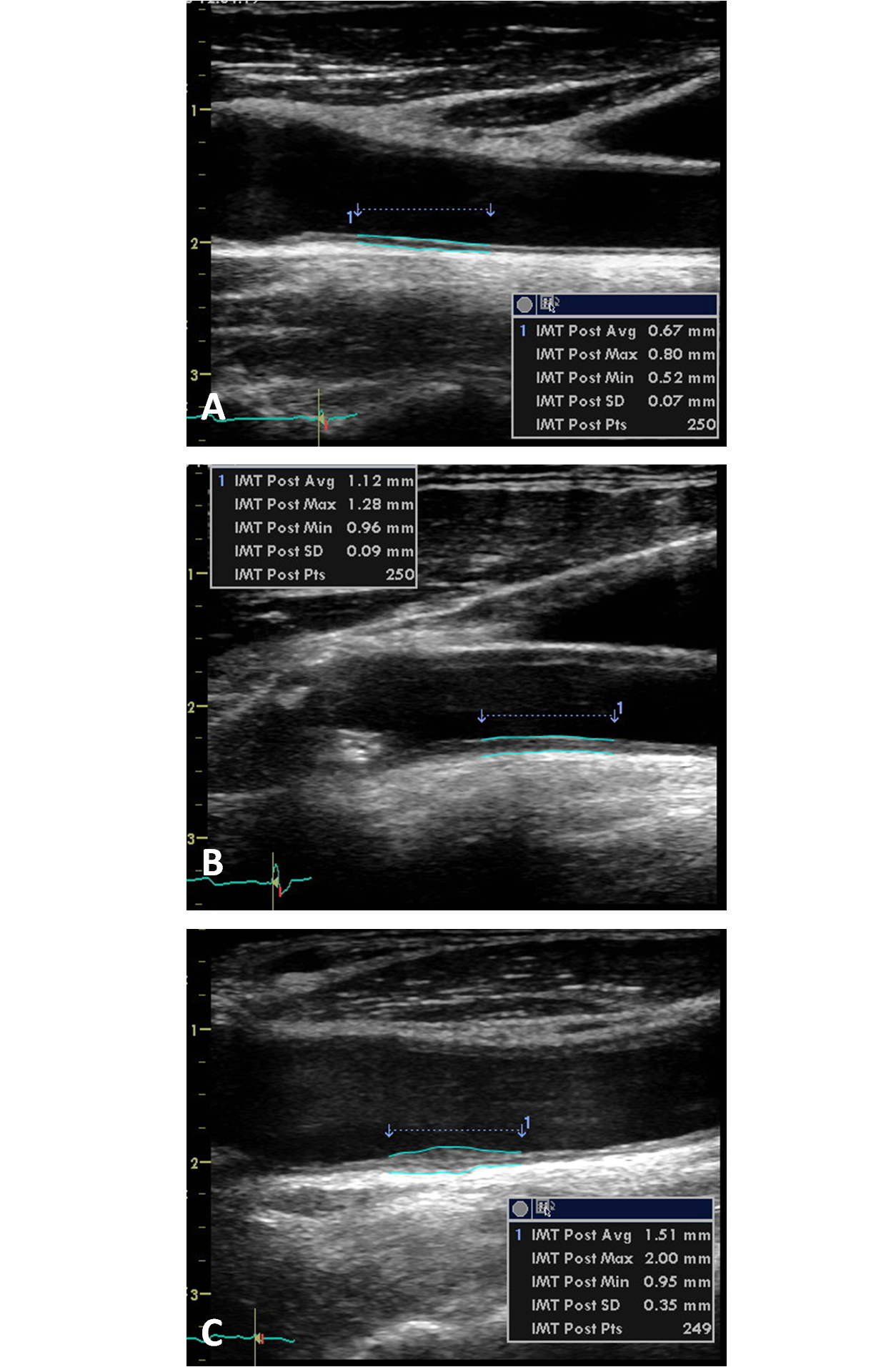
**

**Supplemental Figure 2:** Examples of CIMT measurement: A- normal CIMT, B- increased CIMT, C- carotid artery plaque within the detecting area of CIMT).

**Supplemental Table 1: Intrareader and interreader concordance of carotid intima-media thickness and carotid artery plaque assessment (4 reader, n= 60 subjects, 120 carotid arteries)**

|  | CIMTmean | CIMTmax | CCA plaque | Bulb plaque | Carotid plaque score |
| --- | --- | --- | --- | --- | --- |
| Measure of agreement | CCC | CCC | Alpha | Alpha | Alpha |
| Intrareader | 0.95  (0.93-0.96) | 0.91  (0.89-0.93) | 0.79  (0.70-0.86) | 0.88  (0.64-0.91) | 0.90  (0.85-0.93) |
| Interreader | 0.90  (0.85-0.94) | 0.87  (0.81-0.91) | 0.63  (0.46-0.77) | 0.62  (0.49-0.74) | 0.65  (0.52-0.77) |

CIMT – carotid intima-media thickness, CCA common carotid artery, CCC – concordance correlation coefficient, alpha – Krippendorff’s alpha.

**Supplemental Table 2: Factors to predict revascularization obtained from logistic regression**

| **Variable** | **Unadj. Odds Ratio (95% CI)** | **P-Value** | **Adj. Odds Ratio (95% CI)** | **P-Value** |
| --- | --- | --- | --- | --- |
| ***Traditional risk factors*** |  |  |  |  |
| Age, per 5y increase | 1.08 (1.04-1.13) | <0.001 |  |  |
| Male sex | 2.28 (1.86-2.79) | <0.001 |  |  |
| Diabetes | 1.30 (1.08-1.57) | <0.001 |  |  |
| Dyslipidemia | 2.01 (1.67-2.41) | <0.001 |  |  |
| Hypertension | 1.05 (0.82-1.34) | 0.700 |  |  |
| Tobacco use - Former | 1.46 (1.19-1.78) | <0.001 |  |  |
| Current | 1.55 (1.20-2.00) | 0.001 |  |  |
| ***Additional clinical characteristics*** | | | | |
| Family history of CAD | 1.10 (0.89-1.36) | 0.386 | 1.18 (0.95-1.48) | 0.137 |
| LV-EF <50% | 1.69 (1.33-2.14) | <0.001 | 1.34 (1.04-1.73) | 0.023 |
| hsCRP, per 5mg/l | 1.10 (1.05-1.15) | <0.001 | 1.08 (1.02-1.13) | 0.003 |
| Nt-proBNP, per 500 ng/l | 1.09 (1.05-1.14) | <0.001 | 1.06 (1.02-1.10) | 0.003 |
| ***Clinical presentation*** |  |  |  |  |
| Nonanginal chest pain | 0.76 (0.56-1.03) | 0.078 | 0.91 (0.66-1.25) | 0.566 |
| Atypical angina | 1.31 (1.03-1.68) | 0.028 | 1.62 (1.25-2.10) | <0.001 |
| Typical angina | 2.67 (2.12-3.37) | <0.001 | 3.34 (2.60-4.27) | <0.001 |
| Dyspnea NYHA II | 0.81 (0.67-0.99) | 0.034 | 0.88 (0.72-1.08) | 0.214 |
| NYHA III | 0.69 (0.50-0.94) | 0.019 | 0.69 (0.50-0.97) | 0.032 |
| ***Noncoronary atherosclerosis*** | | | | |
| History of PVD | 2.61 (1.73-3.91) | <0.001 | 1.95 (1.27-2.98) | 0.002 |
| Carotid artery plaque | 3.31 (2.72-4.04) | <0.001 | 2.61 (2.11-3.22) | <0.001 |
| 1 segment | 1.95 (1.48-2.57) | <0.001 | 1.70 (1.28-2.26) | <0.001 |
| 2 segments | 3.15 (2.45-4.04) | <0.001 | 2.59 (1.99-3.37) | <0.001 |
| 3 segments | 4.83 (3.56-6.58) | <0.001 | 3.80 (2.74-5.27) | <0.001 |
| 4 segments | 6.06 (4.38-8.40) | <0.001 | 4.46 (3.15-6.31) | <0.001 |
| CIMTmean per 0.1mm | 1.21 (1.14-1.29) | <0.001 | 1.08 (1.00-1.16) | 0.038 |
| CIMTmax per 0.1mm | 1.19 (1.13-1.26) | <0.001 | 1.08 (1.02-1.15) | 0.013 |
| ABI 1.0-1.09 | 1.06 (0.86-1.32) | 0.575 | 1.12 (0.89-1.40) | 0.328 |
| 0.9-0.99 | 1.96 (1.40-2.75) | <0.001 | 2.00 (1.40-2.85) | <0.001 |
| 0.5-0.89 | 2.73 (2.02-3.70) | <0.001 | 2.41 (1.73-3.34) | <0.001 |
| <0.5 | 7.48 (3.92-14.3) | <0.001 | 6.16 (3.14-12.1) | <0.001 |
| ABI<1.0 | 2.62 (2.11-3.26) | <0.001 | 2.35 (1.85-2.98) | <0.001 |
| NCA | 3.66 (2.97-4.50) | <0.001 | 2.96 (2.37-3.69) | <0.001 |

**Supplemental Table 3: Test performance of carotid artery plaque and NCA to predict prevalent obstructive CAD and the intention for coronary revascularization**

| **Measure** | **Carotid artery plaque** | | **NCA** | |
| --- | --- | --- | --- | --- |
| **Outcome** | **Obstructive CAD** | **Revascularization** | **Obstructive CAD** | **Revascularization** |
| Total cohort |  |  |  |  |
| Sensitivity | 74 (71-77) | 75 (71-78) | 78 (75-81) | 79 (76-82) |
| Specificity | 57 (54-59) | 53 (50-55) | 53 (50-55) | 49 (47-51) |
| PPV | 55 (52-57) | 42 (40-45) | 54 (51-57) | 42 (39-45) |
| NPV | 76 (73-78) | 82 (79-84) | 78 (75-80) | 84 (81-86) |
| Subjects with atypical clinical presentation | | | | |
| Sensitivity | 72 (68-75) | 73 (69-77) | 77 (74-81) | 79 (75-83) |
| Specificity | 57 (54-59) | 54 (51-56) | 53 (50-55) | 50 (47-52) |
| PPV | 48 (45-51) | 35 (33-39) | 48 (45-51) | 36 (33-39) |
| NPV | 78 (76-81) | 85 (82-87) | 81 (79-84) | 87 (84-89) |

Sensitivity, specificity, positive predictive value (PPV) and negative predictive value (NPV) are given in percentage (95% confidence interval).

**Supplemental Table 4: Comparing probabilities of CAD/revascularization in patients without and with noncoronary atherosclerosis**

|  | **No or**  **Nonanginal Chest Pain** | | **Atypical Angina** | | **Typical Angina** | |
| --- | --- | --- | --- | --- | --- | --- |
|  | **Men** | **Women** | **Men** | **Women** | **Men** | **Women** |
| **Obstructive CAD** | | | | | | |
| <60y | 21/48 | 10/28 | 15/50 | 9/39 | 38/78 | 27/40 |
| ≥60y | 26/50 | 14/36 | 39/68 | 18/36 | 68/79 | 15/57 |
| **Coronary Revascularization** | | | | | | |
| <60y | 15/37 | 9/16 | 14/42 | 7/32 | 34/64 | 27/37 |
| ≥60y | 15/36 | 7/25 | 27/55 | 13/30 | 55/65 | 9/46 |

We present percentages with obstructive CAD or coronary revascularization. The first value is the percentage for a patient without evidence of NCA. The second is that of a patient with the presence of NCA (carotid artery plaque or ABI<1.0 or known peripheral artery disease).

Reference

1. Stein JH, Korcarz CE, Hurst RT, Lonn E, Kendall CB, Mohler ER, Najjar SS, Rembold CM, Post WS. Use of carotid ultrasound to identify subclinical vascular disease and evaluate cardiovascular disease risk: A consensus statement from the american society of echocardiography carotid intima-media thickness task force. Endorsed by the society for vascular medicine. *Journal of the American Society of Echocardiography : official publication of the American Society of Echocardiography*. 2008;21:93-111; quiz 189-190
